# Crystallization of Supercooled Liquids: Self-Consistency Correction of the Steady-State Nucleation Rate

**DOI:** 10.3390/e22050558

**Published:** 2020-05-16

**Authors:** Alexander S. Abyzov, Jürn W. P. Schmelzer, Vladimir M. Fokin, Edgar D. Zanotto

**Affiliations:** 1 National Science Center, Kharkov Institute of Physics and Technology, 61108 Kharkov, Ukraine; alexander.abyzov@gmail.com; 2 Institut für Physik der Universität Rostock, Albert-Einstein-Strasse 23-25, 18059 Rostock, Germany; 3 Vitreous Materials Laboratory, Department of Materials Engineering, Federal University of São Carlos, UFSCar, 13565-905 São Carlos-SP, Brazil; vmfokin@gmail.com (V.M.F.); dedz@ufscar.br (E.D.Z.)

**Keywords:** nucleation, crystal growth, general theory of phase transitions, 64.60.Bd General theory of phase transitions, 64.60.Q- Nucleation, 81.10.Aj Theory and models of crystal growth

## Abstract

Crystal nucleation can be described by a set of kinetic equations that appropriately account for both the thermodynamic and kinetic factors governing this process. The mathematical analysis of this set of equations allows one to formulate analytical expressions for the basic characteristics of nucleation, i.e., the steady-state nucleation rate and the steady-state cluster-size distribution. These two quantities depend on the work of formation, $\Delta G\left(n\right)=-n\Delta \mu +\gamma n^{2/3}$, of crystal clusters of size *n* and, in particular, on the work of critical cluster formation, $\Delta G(n_{c})$. The first term in the expression for ΔG(n) describes changes in the bulk contributions (expressed by the chemical potential difference, Δμ) to the Gibbs free energy caused by cluster formation, whereas the second one reflects surface contributions (expressed by the surface tension, σ: $\gamma =\Omega d_{0}^{2}\sigma$, $\Omega =4\pi {\left(3/4\pi \right)}^{2/3}$, where $d_{0}$ is a parameter describing the size of the particles in the liquid undergoing crystallization), *n* is the number of particles (atoms or molecules) in a crystallite, and $n=n_{c}$ defines the size of the critical crystallite, corresponding to the maximum (in general, a saddle point) of the Gibbs free energy, *G*. The work of cluster formation is commonly identified with the difference between the Gibbs free energy of a system containing a cluster with *n* particles and the homogeneous initial state. For the formation of a “cluster” of size n=1, no work is required. However, the commonly used relation for ΔG(n) given above leads to a finite value for n=1. By this reason, for a correct determination of the work of cluster formation, a self-consistency correction should be introduced employing instead of ΔG(n) an expression of the form $\Delta \tilde{G}\left(n\right)=\Delta G\left(n\right)-\Delta G\left(1\right)$. Such self-consistency correction is usually omitted assuming that the inequality ΔG(n)≫ΔG(1) holds. In the present paper, we show that: (i) This inequality is frequently not fulfilled in crystal nucleation processes. (ii) The form and the results of the numerical solution of the set of kinetic equations are not affected by self-consistency corrections. However, (iii) the predictions of the analytical relations for the steady-state nucleation rate and the steady-state cluster-size distribution differ considerably in dependence of whether such correction is introduced or not. In particular, neglecting the self-consistency correction overestimates the work of critical cluster formation and leads, consequently, to far too low theoretical values for the steady-state nucleation rates. For the system studied here as a typical example (lithium disilicate, ${\mathrm{Li}}_{2}O\cdot 2{\mathrm{SiO}}_{2}$), the resulting deviations from the correct values may reach 20 orders of magnitude. Consequently, neglecting self-consistency corrections may result in severe errors in the interpretation of experimental data if, as it is usually done, the analytical relations for the steady-state nucleation rate or the steady-state cluster-size distribution are employed for their determination.

## 1. Introduction

The properties of polycrystalline materials are determined by their chemical composition and volume fraction, shape, size distribution, orientation, and degree of dispersion of the different crystalline phases formed during fabrication [1,2,3,4,5]. Crystallization processes are of particular importance in glass technology, where the rates of crystal nucleation and growth decide whether a given liquid can be vitrified or is likely to crystallize on the cooling path [6,7,8,9,10,11,12]. Indeed, as mentioned long ago, in 1938, by Morey [13]: “Devitrification is the chief factor which limits the composition range of practical glasses, and it is an ever-present danger in all glass manufacture and working, and takes place promptly with any error in composition or technique”. Crystallization plays a major role also in a variety of biological, geological, and other processes occurring in nature, so they surround us in every-day life [14,15,16,17,18,19].

To understand the type of evolution and to control the properties of the newly evolving crystalline phases in an undercooled liquid, as well as the characteristics of the resulting material, an in-depth knowledge of the mechanisms of nucleation and crystal growth is required [2,4,5,9,10,15,16]. Hereby, both general thermodynamic aspects and the details of the kinetics of aggregation of ambient phase particles to the newly evolving crystalline phase are of fundamental importance. In the theoretical analysis of these problems, several problems remain unsolved [20,21,22,23,24,25,26,27]. Here, we will consider in detail one particular issue that was already formulated in the first stages of development of the classical theory of nucleation and growth processes (CNT) [28], but has been widely neglected in applications. It can be described as follows.

In the analysis of thermodynamic aspects of phase formation, in general, and crystallization processes, in particular, different approaches have been employed (e.g., [29,30,31,32,33,34,35]) following and advancing the two alternative methods of theoretical description of thermodynamically heterogeneous systems developed by J. W. Gibbs [36,37] and J. D. van der Waals [38,39]. In application to nucleation, both methods deal with the appropriate description of the work of critical cluster formation, $W_{c}$, i.e., the work to be performed in a reversible process to create a cluster of the new phase that is capable of further deterministic growth. In line with the statistical theory of fluctuation processes [40,41,42], the rate of formation of such critical clusters per unit time in a unit volume of a given ambient phase, $J_{st}$, can be expressed in the form:

(1) $$J_{st}=J_{0}\exp\left(-\frac{W_{c}}{k_{B}T}\right)\;,$$

as first suggested by Volmer and Weber [43]. Here, $k_{B}$ is the Boltzmann constant and *T* the absolute temperature. The pre-exponential term, $J_{0}$, reflects the kinetics of aggregation processes described, in particular, by the appropriate diffusion coefficient. Thermodynamic aspects are mainly represented in Equation (1) by the work of critical cluster formation. This quantity is of essential significance for the correct description of nucleation processes and will be discussed here first.

In the case of homogeneous nucleation of spherical nuclei, the size (radius) of the critical cluster, $R_{c}$, its surface area, $A_{c}$, and the work of critical cluster formation, $W_{c}$, are given by the following relations [2,4,5,35,44]:

(2) $$R_{c}=\frac{2\sigma }{\Delta g}\;,\;W_{c}=\frac{1}{3}\sigma A_{c}=\frac{16\pi }{3}\frac{\sigma^{3}}{{\left(\Delta g\right)}^{2}}\;,\;A_{c}=4\pi R_{c}^{2}$$

or, equivalently, by:

(3) $$R_{c}=\frac{2\sigma }{c\Delta \mu }\;,\;W_{c}=\frac{16\pi }{3}\frac{\sigma^{3}}{{\left(c\Delta \mu \right)}^{2}}\;,\;c\Delta \mu =\Delta g\;.$$

Here, Δg is the difference in the Gibbs free energy per unit volume of the liquid and the crystalline phases and Δμ is the difference in the chemical potential per particle in the liquid and the crystal. Moreover, σ is the supercooled liquid/nucleus surface tension (referred to the surface of tension [36,37]), and *c* is the particle number density of the basic units of the ambient phase. Possible effects of elastic stresses on crystallization are neglected here. Their effect on crystal nucleation can be easily incorporated into the theory as described in detail in [4,5,45,46,47,48,49,50]. The account of elastic stresses leads, as a rule, merely to a change of the values of some parameters in the theoretical description without affecting the main results of the present study.

We consider here crystallization at a given pressure and temperature. For this reason, the appropriate thermodynamic potential for the description of the state of the system is the Gibbs free energy, and the work of critical cluster formation is equal to the change of the Gibbs free energy caused by the formation of a crystallite of critical size, i.e., $W_{c}=\Delta G_{c}$. We assume here that the critical clusters have a spherical shape. The possibility of such treatment was justified in [34,35]. Moreover, we treat phase formation in one-component systems. However, the results also hold for multi-component systems (both for stoichiometric and non-stoichiometric crystallization) as far as one main standard assumption of CNT is fulfilled, i.e., that the bulk properties of the clusters of any size are identical to the properties of the newly evolving macroscopic phases [4,5,35,51,52,53,54,55]. Indeed, as far as the bulk properties of the clusters are defined, one parameter specifying the size of the clusters is sufficient to complete their description. Note that the bulk properties of sub-, critical, and super-critical clusters may deviate from the properties of the evolving macroscopic phases [4,5,9,56]. Their dependence on cluster size can be determined based on the generalized Gibbs’ approach [21,22,23,24,25]. Once this information is obtained, one parameter is, again, sufficient for the description of the state of the evolving crystallites, and the analysis can be performed in a similar way as described here. Examples of such more general treatment in solving the set of kinetic equations of CNT were given in [57,58]. However, to concentrate the attention on the main topic of our analysis, we use here the common approach of CNT leaving the mentioned generalization for future studies.

The change of the Gibbs free energy in cluster formation per unit volume of the crystalline phase can be generally expressed as [4,5,44]:

(4) $$\Delta g=s_{\alpha }\left(T_{\beta }-T_{\alpha }\right)+\left(p_{\alpha }-p_{\beta }\right)+\sum_{i=1}^{k}\rho_{i\alpha }\left(\mu_{i\beta }\left(T_{\beta },p_{\beta },\left\{x_{j\beta }\right\}\right)-\mu_{i\alpha }\left(T_{\alpha },p_{\alpha },\left\{x_{j\alpha }\right\}\right)\right)\;.$$

Here, *s* is the entropy per unit volume, *p* the pressure, *T* the temperature, and $\rho_{i}$, $x_{i}$, and $\mu_{i}$ the densities, molar fractions, and chemical potentials of the different components in the ambient liquid (specified by β) and the newly evolving crystalline (specified by α) phases. By a Taylor expansion of the chemical potentials of the cluster phase with respect to temperature and pressure and adopting the standard assumption of CNT that the composition of the newly evolving phase is equal to the composition of the respective macroscopic phase, Equation (4) yields:

(5) $$\Delta g\left(T,p\right)≅\sum_{i=1}^{k}\rho_{i\alpha }\left(\mu_{i\beta }\left(T,p,\left\{x_{j\beta }\right\}\right)-\mu_{i\alpha }\left(T,p,\left\{x_{j\alpha }\right\}\right)\right)\;.$$

Here, we denote by *T* and *p* the temperature and pressure in the liquid (the ambient phase in the considered case). We consider crystallization at constant pressure. We describe the temperature dependence of the thermodynamic driving force by the approximate relation:

(6) $$\Delta g\left(T\right)≅\Delta h_{m}\left(\frac{T_{m}-T}{T_{m}}\right)\left[1-\frac{\Delta c_{p}}{\Delta s_{m}}\frac{\left(T_{m}-T\right)}{2T_{m}}\right]\;,$$

which is a direct consequence of Equation (5) [4,5,35,44]. Here, $\Delta h_{m}$ and $\Delta s_{m}$ are the melting enthalpy and melting entropy per unit volume of the crystal phase ($\Delta h_{m}=T_{m}\Delta s_{m}$), and $\Delta c_{p}$ is the difference in the specific heats per unit volume of the different phases, both taken at the melting or liquidus temperature, $T_{m}$, and the respective value of pressure, $p_{m}$.

The pre-exponential term, $J_{0}$, in Equation (1) can be determined using the basic model of CNT, assuming that crystallization proceeds by aggregation and dissolution of single particles to/from a crystallite. This model was first formulated by Farkas [59] (with reference to Leo Szilard) and utilized for the derivation of Equation (1) and the specification of $J_{0}$. This work of Farkas was extended by Volmer et al. (see [60]), Kaischew and Stranski [61], Becker and Döring [62], and later, by Frenkel [63], Zeldovich [64], Turnbull and Fisher [65], and others.

In the mathematical development of this model, not only the work of formation of critical clusters has to be known, but also of clusters of arbitrary sizes with radius, *R*, or number of particles, *n*. In application to crystal nucleation, the relations:

(7) $$\Delta G\left(R\right)≅-V\Delta g+\sigma A\;,\;V=\frac{4\pi }{3}R^{3}\;,\;A=4\pi R^{2}$$

or:

(8) $$\Delta G\left(n\right)≅-n\Delta \mu +\gamma n^{2/3}\;,\;\gamma =\Omega d_{0}^{2}\sigma \;,\;\Omega =4\pi {\left(\frac{3}{4\pi }\right)}^{2/3}$$

are employed (e.g., [2,4,5,44,60]), where $d_{0}$ is a characteristic size parameter determined by the particle number density ($c=(1/d_{0}^{3})$). Details of the derivation of these relations and some specific problems in their application were discussed in more detail in [66,67,68,69]. As far as the above noted standard assumption of CNT is fulfilled, these relations can be considered as a sufficiently correct approximation for both one-component and multi-component systems. The advantage of these approximations, Equations (5)–(8), is that the thermodynamic driving force of cluster formation, Δg or Δμ, is independent of cluster size. For this reason, these relations and their consequences can be easily employed in the numerical computations, as we will describe later.

In terms of particle numbers, the correct expressions for the critical cluster size and the work of critical cluster formation are given similarly to Equations (2) and (3) by:

(9) $$\Delta G\left(n_{c}\right)=\frac{1}{2}n_{c}\Delta \mu \;,\;n_{c}^{1/3}=\frac{2\gamma }{3\Delta \mu }\;.$$

However, searching for the extremum of ΔG utilizing the approximate relations, Equation (7) or (8), as the starting point, slightly different relations (as compared to Equations (2), (3) and (9)) are obtained when a curvature dependence of the surface tension is accounted for. The specification of the dependence of the surface tension on temperature, pressure, and/or the size of the clusters employed in the computations and some consequences will be discussed in detail in Section 3.2.

Using Equation (7) and employing the capillarity approximation, we get a dependence for the work of formation, ΔG(R), of a cluster of size, *R*, of the form:

(10) $$\frac{\Delta G(R)}{\Delta G(R_{c})}=3{\left(\frac{R}{R_{c}}\right)}^{2}-2{\left(\frac{R}{R_{c}}\right)}^{3}\;.$$

**Figure 1:**
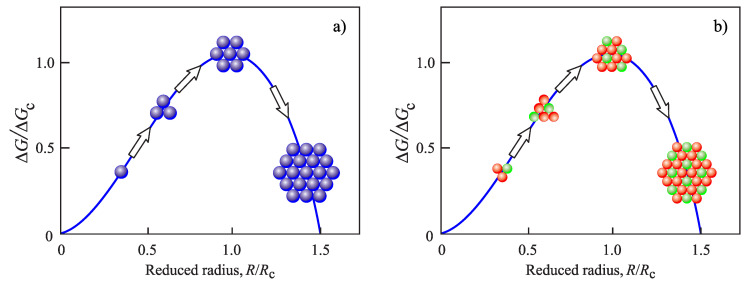
Standard model of the theory of crystal nucleation in classical nucleation theory. The work of critical cluster formation, given by Equation (2), is commonly interpreted as the work required to form a cluster of size $R_{c}$, starting with a size R=0. The evident fact that the change of the cluster radius starts at the size of the atoms or molecules, $R_{atom}$, forming the clusters and not at R=0 is considered to be of minor importance and neglected in the computations. We consider here one-component systems (**a**). However, as far as the standard assumption of CNT employed, i.e., the bulk properties of the crystallites do not depend on their sizes (**b**), the method is equally applicable to both stoichiometric and noon-stoichiometric nucleation in multi-component systems (see the text for a more detailed discussion).

It is illustrated in Figure 1. This dependence is only slightly modified, if a curvature dependence of the surface tension is accounted for. The work of critical cluster formation is commonly interpreted as the work required to form a cluster of size, $R_{c}$, starting with a size R=0. Of course, it is evident that the change of the radius starts at the size of the atoms or molecules forming the clusters. Therefore, in terms of radii, the change of ΔG starts at the radius of the atom or molecule denoted as $R_{atom}$. Using this notation, the work required to form the critical cluster should be written (more correctly) as:

(11) $${\tilde{W}}_{c}=\Delta G\left(R_{c}\right)-\Delta G\left(R_{atom}\right)=\frac{1}{3}\sigma A_{c}-\Delta G\left(R_{atom}\right)\;.$$

However, it is frequently assumed in applications that the account of such correction leads to negligible changes of the main results.

Furthermore, from a formal point of view, a close look at Equations (1)–(9) reveals some inconsistency in the mathematical relations. Monomers (atoms or molecules) exist in the system from the very beginning of the process of phase formation considered. For their formation, no work or change of the Gibbs free energy is required. However, according to Equation (8), ΔG(1) is not equal to zero. In terms of particle numbers, a more correct treatment would be to account, consequently, only for the work required to form a crystallite starting with a “cluster” of size n=1. For the work of formation of a cluster consisting of *n* particles, instead of ΔG(n), the quantity $\Delta \tilde{G}\left(n\right)$ should be employed, consequently, defined via:

(12) $$\Delta \tilde{G}\left(n\right)=\Delta G\left(n\right)-\Delta G\left(1\right)\;.$$

This problem was analyzed in detail by Wu [70], who also provided an overview on earlier attempts of introduction of a “self-consistency correction” (as this topic is denominated) devoted to vapor condensation. It was addressed briefly later by Ford [71] and in more detail by Kelton and Greer [2]. In numerical computations, the problem with the correct value of ΔG for n=1 can be partly resolved by setting ΔG(1)=0 and employing Equation (8) only for n≥2 as done, for example, in [72]. Self-consistency corrections have, of course, negligible effects on the value of the steady-state nucleation rate, Equation (1), if $\Delta G\left(1\right)\ll \Delta G\left(n_{c}\right)$ holds. It is commonly assumed in the interpretation of experimental data based on Equation (1) that this inequality is fulfilled. However, is this really always the case, and does the mentioned common neglect of self-consistency corrections really lead to accurate results?

**Figure 2:**
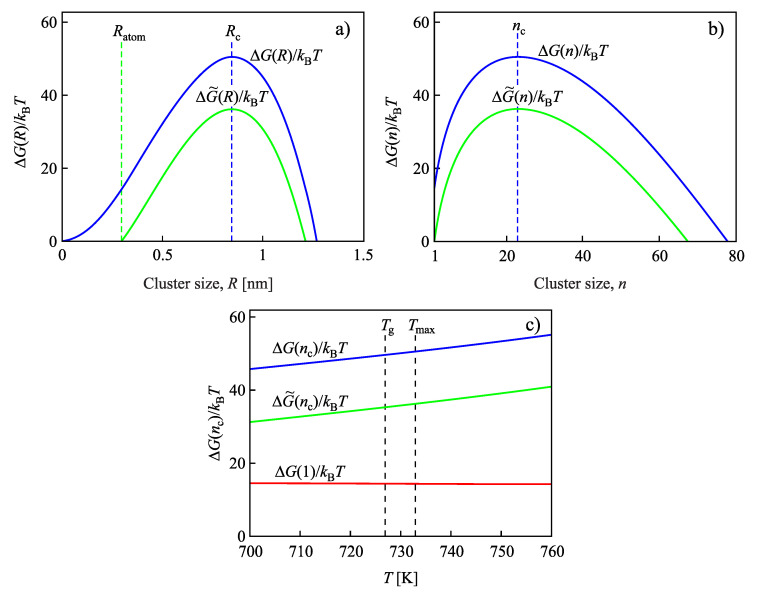
Work of cluster formation (divided by $k_{B}T$) for crystallization of lithium disilicate, ${\mathrm{Li}}_{2}O\cdot 2{\mathrm{SiO}}_{2}$ (L2S), as a function of cluster size and temperature: (**a**) ΔG(R) and $\Delta \tilde{G}\left(R\right)$ versus *R* at T=733 K. ΔG(R) is given by Equation (7), and $\Delta \tilde{G}\left(R\right)$ is defined in analogy to Equation (11) as $\Delta \tilde{G}\left(R\right)=\Delta G\left(R\right)-\Delta G\left(R_{atom}\right)$. (**b**) ΔG(n) and $\Delta \tilde{G}\left(n\right)$ versus *n* at T=733 K; ΔG(n) and $\Delta \tilde{G}\left(n\right)$ are computed via Equations (8) and (12), correspondingly, and ΔG(1) is obtained from Equation (8) setting *n* equal to n=1. (**c**) Work of critical cluster formation, $\Delta G_{c}\left(T\right)$, $\Delta {\tilde{G}}_{c}\left(T\right)$, and the self-consistency correction, ΔG(1), for L2S as a function of temperature. The thermodynamic driving force is computed via Equation (6) with $T_{m}=1307$ K, $\Delta h_{m}=9.744\times 10^{8}$ J/m${}^{3}$, $\Delta c_{p}/\Delta s_{m}=0.275$, $d_{0}=4.8\times 10^{-10}$ m [73,74]. These expressions and parameter values are also used in the subsequent figures. The surface tension is taken as $\sigma =0.170\;J/m^{2}$. $R_{atom}$ is defined as the radius of the atoms or molecules of the liquid undergoing crystallization.

An illustration of these problems and a first answer to the latter questions is given in Figure 2. As an experimental example, we treat here crystallization of lithium disilicate, ${\mathrm{Li}}_{2}O\cdot 2{\mathrm{SiO}}_{2}$ (L2S) [73,74]. The thermodynamic driving force is computed via Equation (6) with $T_{m}=1307$ K, $\Delta h_{m}=9.744\times 10^{8}$ J/m${}^{3}$, and $\Delta c_{p}/\Delta s_{m}=0.275$; to the surface tension, the value $\sigma =0.170\;J/m^{2}$ is assigned; the parameter $d_{0}$ is specified as $d_{0}=4.8\times 10^{-10}$ m. In the figure, both functions ΔG(R) and $\Delta \tilde{G}\left(R\right)$, respectively, ΔG(n) and $\Delta \tilde{G}\left(n\right)$ are shown in dependence on cluster size, *R* or *n*. Note that these functions are merely shifted along the ordinate axis, retaining the value of the critical cluster size, $R_{c}$ or $n_{c}$, unchanged. However, the shift in the ordinate axis is significant, leading to considerable variations of the work of cluster formation, in general, and the work of critical cluster formation, in particular.

Considering phase formation in a selected glass-forming melt, we demonstrate with the results presented in this figure that the inequality $\Delta G\left(1\right)\ll \Delta G\left(n_{c}\right)$ is not generally fulfilled. Since both the system of kinetic equations modeling nucleation and growth and the analytical expressions for the steady-state nucleation rate and the steady-state cluster-size distribution depend on the work of cluster formation, self-consistency corrections can be expected to have a significant effect on the theoretical predictions. In particular, we will show that the results obtained for the mentioned basic characteristics of nucleation by the solution of the set of kinetic equations modeling crystal nucleation and growth may differ considerably from results obtained based on the application of the analytical relations if self-consistency corrections are not accounted for. Consequently, the account of self-consistency corrections is required to get equivalent results in both methods of description of the nucleation kinetics and correct results in their application to the description of experimental data. We will show why the mentioned internal contradiction in the theoretical treatment occurs. As it turns out, the results of the numerical solution of the basic set of kinetic equations are not affected by self-consistency corrections. The origin of such independence will be explained. However, the theoretical predictions of the analytical relations both for the steady-state nucleation rate and the steady-state cluster-size distribution depend significantly on these self-consistency corrections. In particular, neglecting self-consistency correction overestimates the work of critical cluster formation and leads, consequently, to far too low predictions for the steady-state nucleation rates.

The paper is structured as follows: In Section 2, the basic equations for the description of crystal nucleation and growth are formulated in terms of the model advanced by Farkas. They are employed in Section 3 for numerical computation of steady-state nucleation rates and steady-state cluster-size distributions. The results are compared with analytical expressions derived in CNT. In the analysis, we will use first the capillarity approximation (Section 3.1). The same approach will be employed in Section 3.2 accounting appropriately for a temperature or curvature dependence of the surface tension. A discussion of the results and possible further developments, given in Section 4, completes the paper.

## 2. Basic Equations of Classical Nucleation Theory

In the present section, we briefly review the main features of CNT as far as required for the analysis of the problem under consideration. A more detailed description can be found in [4,5] (we mainly rely on it here) or in other references related to similar problems (e.g., [2,10,51,52,53,63,64,65,70,75,76,77]).

As the basic model in CNT, a spatially homogeneous system is considered, where, in the simplest case, particles of one of the components (atoms or molecules) aggregate to form clusters of the new phase. It is supposed that clusters $B_{n}$ consisting of *n* ambient phase molecules (also denoted as particles, monomers, or building units) grow and decay by addition or evaporation of monomers $B_{1}$ only according to the scheme:

(13) $$B_{1}+B_{n}⇌B_{n+1}$$

as in a binary chemical reaction. Such an assumption is quite reasonable in the theoretical treatment of nucleation since the number of monomers exceeds, by many orders of magnitude, the concentration of clusters with particle numbers n>1. Moreover, monomers have the highest mobility. Reactions of other types, as found in coagulation processes [78,79], are excluded in this scheme.

The change of the number of clusters per unit volume, f(n,t), consisting of *n* monomers, with time, *t*, is connected in such a model approach with two possible reaction channels of the form as reflected by Equation (13) involving ($B_{n-1},B_{n},B_{1}$) and ($B_{n},B_{n+1},B_{1}$), respectively. The basic equations for the kinetic description of these processes are given by:

(14) $$\frac{\partial f(n,t)}{\partial t}=J\left(n-1,t\right)-J\left(n,t\right)\;\mathrm{for}\;n\geq 2\;,$$

where the fluxes in cluster-size space are of the form:

(15) $$J\left(n,t\right)=w^{\left(+\right)}\left(n,t\right)f\left(n,t\right)-w^{\left(-\right)}\left(n+1,t\right)f\left(n+1,t\right)\;.$$

Here, $w^{\left(+\right)}\left(n,t\right)$ is the average number of monomers that are incorporated into a cluster of size *n* per unit time, while $w^{\left(-\right)}\left(n,t\right)$ describes the rate of decay processes.

To determine the functions f(n,t) for $n=1,2,\ldots ,n_{max}$ via Equations (14)–(15), the initial and boundary conditions must be specified. In our computations, we use initial conditions of the form:

(16) $$f\left(n,0\right)=c=\frac{1}{d_{0}^{3}}\;.$$

The value of $f(n_{max}+1,t)$ is estimated as:

(17) $$f\left(n_{max}+1,t\right)=f\left(n_{max},t\right)+{\left.\frac{df(n,t)}{dn}\right|}_{n=n_{max}}=2f\left(n_{max},t\right)-f\left(n_{max}-1,t\right)\;,$$

i.e., as the condition of an adsorbing boundary [80,81].

The kinetic coefficients $w^{\left(+\right)}$ and $w^{\left(-\right)}$ have to be determined based on the analysis of the growth and decay kinetics of the clusters. They may differ for each particular system considered, while the general equations, Equations (14) and (15), remain the same. The attachment rates, $w^{\left(+\right)}\left(n,t\right)$, may be determined by a macroscopic approach (see, e.g., [4,5,51,52,53,65,72,75,76,77]). Here, we will employ the expressions derived in [4,5]:

(18) $$w^{\left(+\right)}\left(n,t\right)=\frac{1}{d_{0}^{4}}4\pi R^{2}\left(n\right)D=\frac{k_{B}T}{d_{0}^{5}\eta }4\pi R^{2}\left(n\right)\;,$$

respectively,

(19) $$w^{\left(+\right)}\left(n,t\right)=\Omega \frac{D}{d_{0}^{2}}n^{2/3}=\Omega \frac{k_{B}T}{d_{0}^{3}\eta }n^{2/3}\;.$$

In the right part of Equations (18) and (19), η is the Newtonian viscosity. In their derivation, the Stokes–Einstein–Eyring equation is applied [4,5].

However, in the subsequent computations, we avoid the application of the Stokes–Einstein–Eyring relation. We suppose that the kinetics of aggregation is governed by an appropriately specified diffusion coefficient, *D* [55], written as:

(20) $$D=D_{0}\exp\left(-\frac{E_{D}}{k_{B}T}\right)\;.$$

The activation energy for diffusion, $E_{D}$, depends, in general, on temperature, pressure, and composition of the liquid. Pressure and composition are assumed to be constant and not affected by the phase formation processes considered. A detailed analysis of the influence of depletion effects on nucleation and the whole course of first-order phase transformation can be found in [66,75,76,77,82,83,84].

In the computations performed in the present paper, the diffusion coefficient is determined based on experimental data on the time-lag in nucleation [73,74]. This method is supplemented by other methods of evaluation of the diffusion coefficient from experimental data in order to check its validity. The temperature dependence of the diffusion coefficient for homogeneous crystal nucleation in LS2 is well approximated by Equation (20) employing the following values for $D_{0}$ and $E_{D}$:

(21) $$D_{0}=\left(6.66\times 10^{15}-1.18\times 10^{16}\right)\;m^{2}/s\;,\;E_{D}=8.864\times 10^{-19}\;J\;.$$

These parameter values will be mainly used in the computations. For a direct quantitative comparison with experimental data in wide temperature ranges, we will also employ directly the values of the diffusion coefficient as obtained from time-lag data. The respective procedure will be described later in this article.

While the attachment rates, $w^{\left(+\right)}$, may be determined based on a macroscopic approach, the calculation of the rate of emission of monomers from the cluster requires, in principle, microscopic considerations. Though attempts for a microscopic determination of the detachment rates have been formulated, the most common approach till now remains the application of the principle of detailed balancing involving the so-called equilibrium distribution of the number of clusters as a function of cluster sizes. In such a line of development in the classical derivation of Becker and Döring [62], the rates of evaporation were determined by the Gibbs–Thomson equation. These ideas have been advanced by Frenkel [63] and then utilized generally. However, detailed balancing is valid only for equilibrium states. The so-called equilibrium distributions employed in such an approach are, in general, incorrect for metastable systems. A detailed analysis of this circle of problems can be found in [2,4,5,51,52,53,70,76,77]. However, as shown first by some of us in [51,52,53,85], similar relations connecting the coefficients of aggregation and emission can also be obtained without reference to equilibrium distributions and the principle of detailed balancing. One can derive directly the equations:

(22) $$\frac{w^{\left(+\right)}\left(n,t\right)}{w^{\left(-\right)}\left(n+1\right)}=\exp\left\{-\frac{\Delta G(n+1)-\Delta G(n)}{k_{B}T}\right\}$$

in a form identical to the commonly utilized relations.

As a direct consequence of Equation (22), we obtain the following expression for the steady-state nucleation rate [4,5]:

(23) $$J_{st}=w^{\left(+\right)}\left(n_{c}\right)\Gamma_{Z}c\exp\left(-\frac{\Delta G(n_{c})}{k_{B}T}\right)\;.$$

The Zeldovich factor, $\Gamma_{Z}$, in Equation (23) is defined by:

(24) $$\Gamma_{Z}=\sqrt{-\frac{1}{2\pi k_{B}T}\left({\left.\frac{\partial^{2}\Delta G}{\partial n^{2}}\right|}_{n=n_{c}}\right)}=\frac{1}{2\pi cR_{c}^{2}}{\left(\frac{\sigma }{k_{B}T}\right)}^{1/2}\;.$$

After some straightforward transformations, we arrive with Equation (19) at:

(25) $$J_{st}=c\sqrt{\frac{\sigma }{k_{B}T}}\left(\frac{2D}{d_{0}}\right)\exp\left(-\frac{\Delta G(n_{c})}{k_{B}T}\right)\;.$$

The same result can be obtained by an analysis of the Frenkel–Zeldovich equation giving a continuum description of the processes described here by the above formulated set of kinetic equations [2,4,5,51,52,53,75,76,77,85].

Returning now to the problem with the specification of $\Delta G(n_{c})$ in Equation (25) as discussed in the Introduction, we conclude that Equation (22) can be reformulated as:

(26) $$\frac{w^{\left(+\right)}\left(n,t\right)}{w^{\left(-\right)}\left(n+1\right)}=\exp\left\{-\frac{\Delta \tilde{G}\left(n+1\right)-\Delta \tilde{G}\left(n\right)}{k_{B}T}\right\}\;,$$

with $\Delta \tilde{G}\left(n\right)=\Delta G\left(n\right)-\Delta G\left(1\right)$ (cf. Equation (12)). It follows that the relations between the coefficients of aggregation do not depend on whether self-consistency corrections for ΔG(n) are introduced (Equation (26)) or not (Equation (22)). Consequently, the form of the kinetic equations and the results of computations are not affected by self-consistency corrections. However, the account of a self-consistency correction results in a modification of the analytical relation for the steady-state nucleation rate.

Indeed, as already noted in connection with Figure 2, the shift of the ΔG(n)-curves by such transformation does not lead to a change of the value of the critical cluster size. Consequently, performing now the same computations as in the derivation of Equation (25), we obtain a modified expression for the steady-state nucleation rate:

(27) $$J_{st}=c\sqrt{\frac{\sigma }{k_{B}T}}\left(\frac{2D}{d_{0}}\right)\exp\left(-\frac{\Delta \tilde{G}\left(n_{c}\right)}{k_{B}T}\right)\;.$$

This result is in agreement with the conclusions of Wu [70]; however, as we explained here earlier, it can be obtained without any reference to constraint equilibrium distributions.

As is evident from the above outlined considerations, the procedure in the application of the set of kinetic equations is independent of the inconsistencies mentioned in the Introduction in the specification of the work of cluster formation. It follows that self-consistency corrections are of no relevance if one performs computations based on the set of kinetic equations resulting from the basic model of CNT. Such self-consistency correction does have, however, a direct effect on the analytical expressions obtained for the steady-state nucleation rate. One obtains a different relation, Equation (27), if self-consistency corrections are accounted for as compared with Equation (23), when such corrections are neglected. Consequently, as far as the inequality $\Delta G\left(n_{c}\right)\gg \Delta G\left(1\right)$ does not hold, only one of the analytical equations can coincide with the predictions obtained from the solution of the set of kinetic equations.

In the next section, we will perform a detailed comparison of the results of the numerical solution of the set of kinetic equations, Equations (14) and (15), and their consequences with the analytical expressions for the steady-state nucleation rate, Equations (25) and (27), and discuss in detail some further consequences.

## 3. Comparison of the Different Theoretical Treatments

### 3.1. Application of the Capillarity Approximation

In the application of the basic relations of CNT, expressions for the thermodynamic driving force, Δg, and the surface tension are required. For the specification of Δg, we employ here Equation (6). As the first and simplest assumption concerning the surface tension, we assume that the capillarity approximation holds. This is another assumption widely employed in CNT. It presumes that the surface tension of critical clusters is equal to that for an equilibrium coexistence of liquid and crystal at planar interfaces at the given pressure, $p_{m}$, and the corresponding melting temperature, $T_{m}$. In application of the analytical expressions for the steady-state nucleation rates, this assumption refers to clusters of critical sizes. In the numerical computations involving Equations (14)–(22), this assumption is extended to clusters of arbitrary sizes.

**Figure 3:**
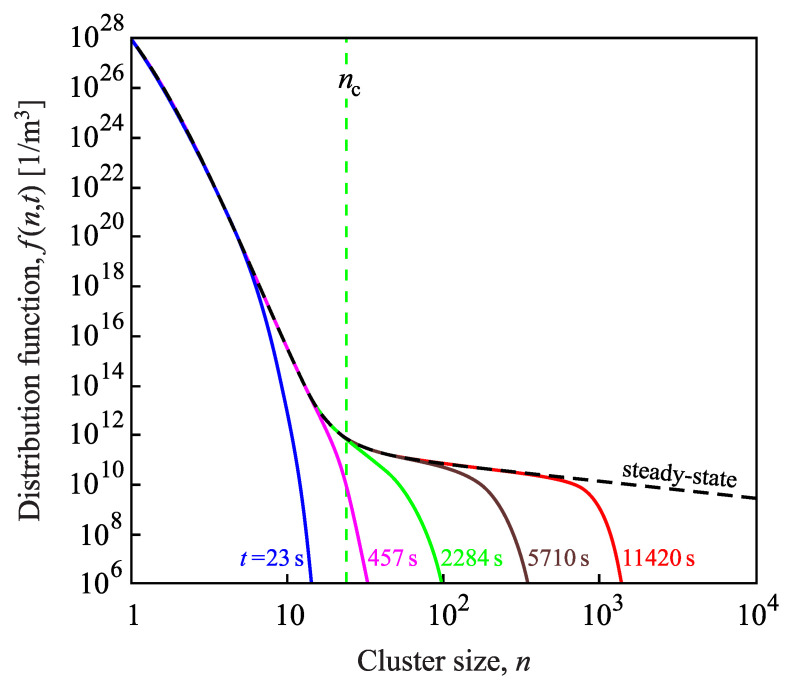
Evolution of the cluster-size distribution function as a function of time at a temperature T=733 K. The diffusion coefficient was also taken to model crystal nucleation in L2S utilizing Equation (20) with $D_{0}=1.18\times 10^{16}\;m^{2}/s$ and $E_{D}=8.864\times 10^{-19}\;J$. The thermodynamic parameters and the value of $d_{0}$ are given in the caption to Figure 2. The dashed curve is computed via the analytical expression, Equation (32), employing the result for the steady-state nucleation rate obtained from the numerical computations.

Results of the solution of the set of kinetic equations utilizing the capillarity approximation are presented in Figure 3 for crystallization of lithium disilicate, ${\mathrm{Li}}_{2}O\cdot 2{\mathrm{SiO}}_{2}$ (L2S). As evident from the figure, in the course of time, a steady-state cluster-size distribution evolves. As soon as steady-state conditions are established for cluster sizes up to $n_{c}$, the steady-state nucleation rate, $J_{st}\left(n_{c}\right)$, can be computed via Equation (15) or equivalent methods.

A theoretical interpretation of such behavior was advanced first by Zeldovich [64]. According to him, a certain time, $\tau_{ns}$, denoted as time-lag in nucleation, is required to establish steady-state nucleation rates and steady-state cluster-size distributions for clusters up to critical sizes in a given system after it has been transferred to the desired metastable initial state. Following Zeldovich, the nucleation rate, $J_{ns}$, in this initial non-steady stage is described as:

(28) $$J_{ns}\left(T,p;t\right)≅J_{st}\left(T,p\right)\exp\left(-\frac{\tau_{ns}}{t}\right)\;.$$

Extensions of the original concepts of Zeldovich were reviewed in [2,4,5,86].

The time-lag is one of the two main parameters that determines the average time, 〈τ〉, required for the formation of a first critical cluster [87],

(29) $$\langle \tau \rangle ≅\tau_{ns}+\frac{1}{J_{st}V}\;,$$

in a system with volume *V*. The time-lag can be estimated as [4,5,79]:

(30) $$\tau_{ns}=\frac{\omega }{2\pi w^{\left(+\right)}\left(n_{c}\right){\left(\Gamma_{Z}\right)}^{2}}\;,$$

where the numerical factor ω varies in the range 1≤ω≤4 depending on the method employed in the derivation of Equation (30). Utilizing Equations (18) and (24) for the specification of the attachment coefficient and the Zeldovich factor, we obtain the following correlation between the diffusion coefficient and time-lag in nucleation:

(31) $$D\left(T\right)=\frac{2\omega \sigma k_{B}T}{{\left(d_{0}\Delta g\right)}^{2}}\left(\frac{1}{\tau_{ns}\left(T\right)}\right)\;.$$

Equation (31) is used directly in the computations and as the starting point for the formulation of the approximative relation, Equations (20) and (21). The parameter ω will be set equal to ω=8/3.

Let us suppose now that such a steady-state distribution has been evolved up to a sufficiently high value, $n_{max}$, of the number of particles in the cluster ($n_{max}\gg n_{c}$) and that $f(n_{max}+1)=0$ holds (Szilard’s model [4,5]). In the whole range of cluster sizes up to $n=n_{max}$, the fluxes obey then the equations $J\left(n\right)=J_{st}\left(n_{c}\right)=$ constant. The set of relations in Equation (15) with $n=1,2,\ldots ,n_{max}$ represents a system of linear equations for the determination of the steady-state values of the functions, $f_{st}\left(n\right)$, $n=1,2,\ldots ,n_{max}$. Employing, in addition, Equation (22), the steady-state cluster-size distribution in this range of cluster sizes can be expressed as [2,81,88]:

(32) $$f_{st}\left(n\right)=J_{st}\sum_{k=n}^{n_{max}}\left(\frac{1}{\omega^{\left(+\right)}\left(k\right)}\right)\exp\left(\frac{\Delta G(k)-\Delta G(n)}{k_{B}T}\right)\;,\;n\leq n_{max}\;.$$

In the range of cluster sizes, $1\leq n\leq n_{c}$, this distribution can be analytically described via the simpler relation [4,5,51,52,53,75,77]:

(33) $$f_{st}\left(n\right)=\left(\frac{c}{2}\right)\exp\left(-\frac{\Delta G(n)}{k_{B}T}\right)\mathrm{erfc}\left(\frac{n-n_{c}}{\delta n_{c}}\right)$$

with:

(34) $$\delta n_{c}=\frac{1}{\sqrt{-\frac{1}{2k_{B}T}\left({\left.\frac{\partial^{2}\Delta G}{\partial n^{2}}\right|}_{n=n_{c}}\right)}}\;.$$

A comparison with Equation (24) shows that $\delta n_{c}$ is directly correlated with the Zeldovich factor.

Both relations, Equations (32) and (33), show that also with respect to the correct expression for the steady-state cluster-size distribution, the question concerning the relevance of introduction of self-consistency corrections occurs. Both equations (Equation (32) via $J_{st}$) depend on the work of formation of a given cluster, consequently, the question is whether either ΔG(n) or $\Delta \tilde{G}\left(n\right)$ has to be substituted into these relations.

In Figure 3, in computing the steady-state cluster-size distribution via Equation (32), we employed the value for the steady-state nucleation rate obtained from the numerical computations. This approach yields a very good agreement between numerical computations and the analytical description by Equation (32). A practically identical curve is obtained if in Equation (32), the value of the steady-state nucleation rate is used, as predicted by Equation (27), where self-consistency corrections are incorporated. This is the first proof that self-consistency corrections are essential to predict the correct values of the steady-state nucleation rates and, consequently, the correct course of the steady-state cluster-size distributions.

**Figure 4:**
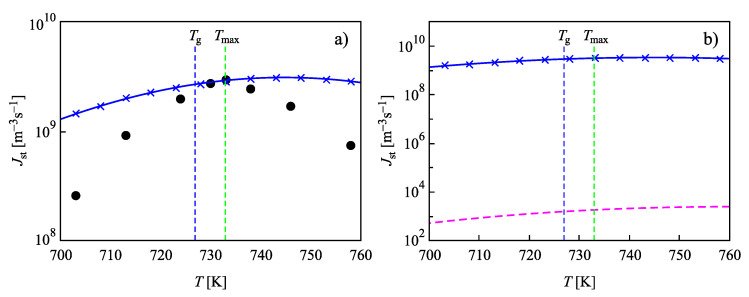
Steady-state nucleation rates for L2S versus temperature: Results are obtained employing the capillary approximation. The full circles in (**a**) show experimental data as reported in [73,74]; data shown by crosses are the numerically calculated nucleation rates obtained by solving the set of kinetic equations, Equations (14)–(22). The surface tension was chosen as $\sigma =0.170\;J/m^{2}$ to yield the best fit of the experimental data at temperatures, $T=T_{max}$, near the maximum of the steady-state nucleation rates. The diffusion coefficient was determined via experimental time-lags [73,74] utilizing Equation (31). The blue line shows the theoretical curve obtained via Equation (27), when self-consistency corrections are accounted for. As is evident, the results of numerical computations and the predictions based on Equation (27) coincide. The dashed line in (**b**) is calculated via the standard approach of CNT, utilizing Equation (25) with the same value of σ. It underestimates the correct value of the steady-state nucleation rate by 7–8 orders of magnitude. By $T_{g}$, the glass transition temperature is specified. The other parameters are given in the captions of Figure 2 and Figure 3.

In Figure 4, the steady-state nucleation rates for L2S versus temperature are shown, computed via the different methods discussed in Section 2. Again, the results are obtained employing the capillary approximation. The full circles in Figure 4a show experimental data as reported in [73,74], and data shown by crosses are the numerically calculated nucleation rates obtained by solving the set of kinetic equations, Equations (14)–(22). The value of the surface tension was chosen as $\sigma =0.170\;J/m^{2}$ to yield the best fit of the experimental data at temperatures, $T=T_{max}$, corresponding to the maximum of the steady-state nucleation. The full blue line shows the theoretical curve obtained via Equation (27), when in the analytical expression, self-consistency corrections are accounted for. By $T_{g}$, the glass transition temperature is specified according to the definition of Tammann [89], who identified the glass transition temperature with values of the Newtonian viscosity equal to $10^{12}$ Pa s. Results of numerical computations and the predictions based on Equation (27) coincide.

The dashed line in Figure 4b is calculated via the standard approach of CNT, utilizing Equation (25) with the same value of σ, but not incorporating self-consistency corrections. It underestimates the correct value of the steady-state nucleation rate by 7–8 orders of magnitude. Consequently, the analytical expressions for the steady-state nucleation rate lead to an agreement with the results based on the computations only when self-consistency corrections are accounted for. This result is in agreement with the general expectations in advancing Equation (1) based on the statistical-mechanical theory of fluctuations as discussed in Section 1. Moreover, it is shown that such self-consistency corrections are, in general, of major importance. If such corrections are omitted, then this may lead in the present case to significant errors up to 7–8 orders of magnitude in the value of the steady-state nucleation rate.

**Figure 5:**
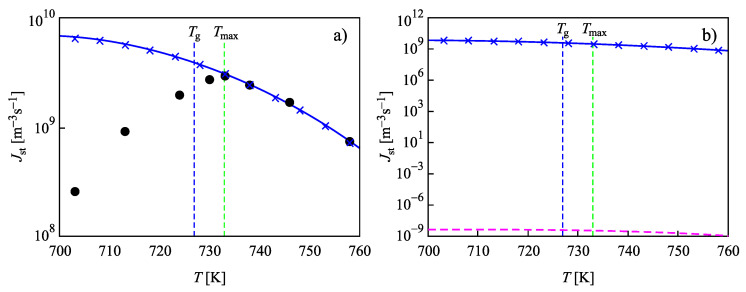
Steady-state nucleation rates for L2S versus temperature: Results are obtained employing the capillary approximation, but for different values of the parameters. The full circles in (**a**) show experimental data as reported in [73,74]. The surface tension was chosen as $\sigma =0.19353\;J/m^{2}$, and $d_{0}$ was set equal to $d_{0}=8.265\times 10^{-10}\;m$. The parameters $D_{0}$ and $E_{D}$ in Equation (20) are $D_{0}=6.66\times 10^{15}\;m^{2}/s$ and $E_{D}=8.864\times 10^{-19}$ J. With these parameters, a fit of the experimental data for the nucleation rates is possible in a wide range of temperatures down to $T≅T_{max}$ near the maximum of the steady-state nucleation. The full blue line shows the theoretical curve obtained via Equation (27), when in the analytical expression self-consistency corrections are accounted for. As is evident, results of numerical computations and the predictions based on Equation (27) coincide. The dashed line in (**b**) is calculated via the standard approach of CNT, utilizing Equation (25) with the same values of σ and $d_{0}$. It is evident that it underestimates the correct value of the steady-state nucleation rate by 18 orders of magnitude. The other parameters are specified in the captions to Figure 2 and Figure 3.

In Figure 4, we used a value of the size parameter $d_{0}$ in line with its definition as a measure of the size of the basic units of the liquid, $c≅1/{\left(d_{0}\right)}^{3}$. The surface tension was chosen as $\sigma =0.1703\;J/m^{2}$ to yield the best fit of the experimental data at temperatures, $T=T_{max}$, near the maximum of the steady-state nucleation. Such an approach, taking the surface tension as a fit parameter, is possible since the values of the surface tension for liquid-crystal equilibrium coexistence even at planar interfaces are not directly measurable with the precision required for the interpretation of experimental data [2,4,5]. As shown in Figure 5, considering both the size parameter and the value of the surface tension as fit parameters, the capillarity approximation may even lead to a coincidence of experimental data and theoretical predictions for the steady-state nucleation rates in wider ranges of temperature.

Indeed, in Figure 5, results for the steady-state nucleation rate with dependence on temperature are presented when the value of the surface tension was chosen as $\sigma =0.19353\;J/m^{2}$ and $d_{0}$ was set equal to $d_{0}=8.265\times 10^{-10}\;m$. The parameters $D_{0}$ and $E_{D}$ in Equation (20) have here the values $D_{0}=6.66\times 10^{15}\;m^{2}/s$ and $E_{D}=8.864\times 10^{-19}$ J. With these parameters, a fit of the experimental data for the nucleation rates is possible in a wide range of temperatures down to $T≅T_{max}$ near the maximum of the steady-state nucleation rate. The full circles in Figure 5a show again experimental data as reported in [73,74]. The full blue line shows the theoretical curve obtained via Equation (27), when in the analytical expression, self-consistency corrections are accounted for. As is evident, the results of numerical computations and the predictions based on Equation (27) coincide. The dashed line in Figure 5b is calculated via the standard approach of CNT, utilizing Equation (25) with the same values of σ and $d_{0}$. It is evident that it underestimates the correct value of the steady-state nucleation rate by 18 orders of magnitude. The other parameters are specified in the captions to Figure 2 and Figure 3.

The mentioned coincidence of theoretical results and experimental data in wide ranges of temperature utilizing the capillarity approximation can be reached only if values are assigned to the parameter $d_{0}$ that do not correspond to its original meaning. Such a procedure can be performed also utilizing Equation (25), neglecting self-consistency corrections. For the particular system considered here, the application of Equation (25) leads to a similarly good fit if the values of the parameters are taken as σ=0.20039 $J/m^{2}$, and $d_{0}=4.8\times 10^{-15}$ m. The latter value of the size parameter does not, however, have any physical meaning.

Similar problems were observed in a variety of applications of CNT when the capillarity approximation was utilized for the interpretation of experimental data [9,50,73,74,90]. In particular, by combining Equations (23) and (30), one can obtain a relation, where the diffusion coefficient is replaced by the time-lag,

(35) $$J_{st}\tau_{ns}=\frac{\omega c}{2\pi \Gamma_{Z}}\exp\left(-\frac{\Delta G(n_{c})}{k_{B}T}\right)\;.$$

Utilizing the expression for the Zeldovich factor, Equation (24), we arrive at:

(36) $$\ln\left(\frac{J_{st}\tau_{ns}{\left(\Delta g\right)}^{2}}{{\left(k_{B}T\right)}^{1/2}}\right)=\ln\left(\frac{4\omega \sigma^{3/2}}{\pi d_{0}^{6}}\right)-\frac{\Delta G(n_{c})}{k_{B}T}\;,$$

respectively,

(37) $$\ln\left(\frac{J_{st}\tau_{ns}{\left(\Delta g\right)}^{2}}{{\left(k_{B}T\right)}^{1/2}}\right)=\ln\left(\frac{4\omega \sigma^{3/2}}{\pi d_{0}^{6}}\right)-\frac{16\pi }{3k_{B}T}\frac{\sigma^{3}}{{\left(\Delta g\right)}^{2}}\;.$$

The so-called “nucleation plot”, generated based on Equation (37), illustrates the dependence of $J_{st}\tau_{ns}{\left(\Delta g\right)}^{2}/{\left(k_{B}T\right)}^{1/2}$ on $1/T{\left(\Delta g\right)}^{2}$. The analysis of the nucleation plot and the relations underlying it leads, for example, for L2S to values of $d_{0}$ of the order of $d_{0}≅10^{-13}$ m, which does not have any physical meaning, and also to highly questionable values of the surface tension [9,50]. As evident from Figure 5, these problems remain, in general, also, if self-consistency corrections are accounted for.

For this reason, we will explore here also the results of the standard approach of CNT in improving the degree of agreement of experimental and theoretical results accounting for a curvature dependence of the surface tension. As shown in previous papers [34,35,49,50,91,92], a correct description of the temperature dependence of the steady-state nucleation rate of crystallites for temperatures above its maximum can be obtained by such an approach. We will extend the present analysis introducing such size or temperature dependence of the surface tension into the description of nucleation accounting appropriately for the self-consistency correction. In connection with the problem under consideration, the main question here is whether self-consistency corrections remain to be of importance if such a temperature or curvature dependence of the surface tension is accounted for or not.

Prior to proceeding in such a way, we have to analyze the question of how a curvature or temperature dependence of the surface tension can be introduced for sub- and super-critical crystallites. The answer will be given based on a brief review on existing results concerning the temperature and/or size dependence of the surface tension of critical crystal clusters in nucleation.

### 3.2. Account of a Temperature or Size Dependence of the Surface Tension

#### 3.2.1. Some General Comments

In the application of relations of the form of Equation (1) in CNT, exclusively expressions for the work of formation of critical clusters are required. For its specification, Gibbs’ theory of heterogeneous systems can be employed dealing also only with the description of equilibrium states. Choosing the surface of tension as the dividing surface, the critical cluster size and the work of critical cluster formation are given by Equation (2) independently of whether a dependence of the surface tension on pressure and/or temperature or curvature is accounted for or not [36,37,66,67,68,69,93,94,95]. Another question is how the dependence of the surface tension of critical clusters on its size can be described.

Equations for the size or curvature dependence of the surface tension of critical clusters have been established by a variety of authors. Some overviews were given in [96,97,98]. Their validity has been checked by the van der Waals method of description of the properties of critical clusters, respectively, more advanced methods of density functional computations. In such an approach, initially, the work of critical cluster formation is established. Then, based on these data, it is specified how the curvature dependence of the surface tension should look like if the results are interpreted in terms of Gibbs’ theory (see, e.g., [35,99,100]).

In recent papers [34,35], the applicability of the Tolman equation [101]:

(38) $$\sigma \left(R_{c}\right)=\frac{\sigma_{\infty }}{1+\frac{2\delta }{R_{c}}}\;,\;\sigma_{\infty }=\sigma \left(R_{c}\to \infty \right)$$

to the description of crystal nucleation was studied in detail. It was shown that: (i) The Tolman equation holds for both one- and multi-component systems, provided the basic assumptions of CNT are fulfilled, if either temperature or pressure are changed. (ii) Gibbs and Tolman analyzed the problem of the curvature dependence of the surface tension of droplets and bubbles in one-component systems assuming that the temperature is kept constant and pressure is changed. Consequently, in [34,35], for the first time, the possibility of the application of the Tolman equation to crystallization was given a sound foundation when crystallization is caused by variations of temperature. (iii) For variations of the degree of metastability caused by variations of pressure, the Tolman parameter is equal to the difference of the radii of two dividing surfaces (its absolute value is, consequently, of the order of magnitude, but not equal to the width of the interface). For temperature-induced crystallization, the Tolman parameter has a different meaning (see [34,35] for a more detailed discussion). (iv) At the same conditions as specified above, if both temperature and pressure are changed, the surface tension depends on both the size of the crystals and the degree of deviation from equilibrium, i.e., the Tolman equation does not hold. (v) Again, at the same conditions as specified above, if only temperature or pressure are varied, then the surface tension is a function of either cluster size or pressure/temperature, but not of both parameters. Critical cluster size and pressure or temperature are directly correlated in such cases. (vi) The Tolman equation can be extended by introducing a more general definition of the Tolman parameter not correlating it with the properties of the interface between liquid and crystal at an equilibrium coexistence of both phases at planar interfaces. The methods of determination of the Tolman parameter both in its original meaning and its generalization are advanced. (vii) For systems with a spinodal curve distinguishing between metastable and thermodynamically unstable initial states (like in condensation and boiling or segregation in solutions), a new relation for the curvature dependence of the surface tension is obtained [35]. It describes its course in the whole range of metastable initial states from the binodal to the spinodal curves. In such cases, the Tolman equation is also not a good approximation. However, as shown in detail in [102], if the basic assumptions of CNT hold, then a spinodal does not exist in melt crystallization. This is one of the major reasons why the Tolman equation allows one a satisfactory description of the size effects in crystal nucleation as shown in [34,35,50,91,92].

All these results refer to the surface tension of critical clusters. However, in the application of Equations (7) and (9), one needs expressions for σ(R) not only for critical, but also for sub- and super-critical clusters. They cannot be obtained directly based on Gibbs’ theory devoted to systems in thermodynamic equilibrium exclusively and also not by van der Waals or more advanced density functional methods dealing with the properties of critical clusters. For this reason, additional approaches or assumptions are required.

#### 3.2.2. Account of a Temperature Dependence of the Surface Tension for Sub- and Super-Critical Clusters

In the numerical computations, we will consider two such assumptions going beyond the studies employing the capillarity approximation, $\sigma =\sigma_{\infty }\left(T_{m},p_{m}\right)$, as discussed in Section 3.1. In the first set of computations presented in this section, we will assume that σ is a function of temperature and has the same value again for clusters of arbitrary sizes. The theoretical results are compared again with experimental data on nucleation rates in dependence on temperature for L2S.

In detail, we proceeded as follows: We assumed in line with our previous investigations that the curvature dependence of the surface tension for the critical clusters, $\sigma (R_{c})$, with radii, $R_{c}\left(T\right)$, can be expressed via the Tolman equation, Equation (38). For any given value of temperature, a particular value of the critical cluster size and a particular value of the surface tension of critical clusters are found. This value of the surface tension is assigned then for this particular temperature to crystallites of arbitrary sizes. In other words, crystallites of any size are assumed to have the same value of the surface tension as the appropriate for this temperature surface tension of critical clusters, $\sigma \left(R\right)=\sigma (R_{c}\left(T\right))=\sigma \left(T\right)$.

**Figure 6:**
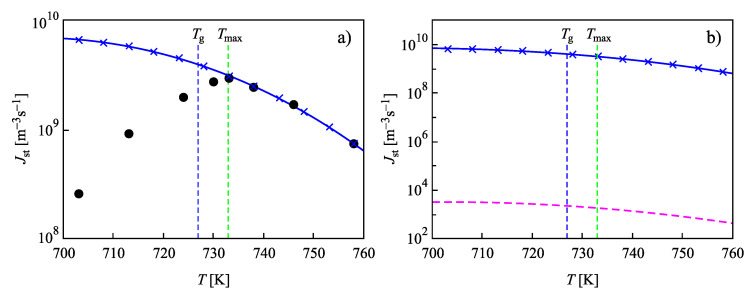
Nucleation rates for L2S versus temperature: Results are obtained by assuming a curvature dependence of the surface tension for the critical clusters, $\sigma (R_{c})$, with radii, $R_{c}\left(T\right)$, expressed by the Tolman equation, Equation (38). In addition, for any temperature, crystallites of any size are assumed to have the same value of the surface tension as the critical clusters, $\sigma \left(R\right)=\sigma (R_{c}\left(T\right))=\sigma \left(T\right)$. The filled circles in (**a**) refer again to experimental data reported in [73,74], whereas the data shown by crosses are the numerically calculated nucleation rates obtained by solving the set of kinetic equations, Equations (14)–(22). The values of the surface tension for a planar coexistence of liquid and crystalline phases of L2S and the Tolman parameter are chosen as $\sigma_{\infty }=0.21178\;J/m^{2}$ and $\delta =0.215d_{0}$ to yield the best fit with the experimental data at temperatures, $T>T_{max}$, above the maximum of the steady-state nucleation rate. The blue line shows the theoretical curve obtained via Equation (27), when self-consistency corrections are accounted for. The diffusion coefficient is described by Equation (20) with $D_{0}=1.18\times 10^{16}\;m^{2}/s$ and $E_{D}=8.864\times 10^{-19}\;J$. The dashed line in (**b**) is calculated, again, via the standard approach of CNT, utilizing Equation (25) with the same values of σ(T). The other parameters are specified in the captions to Figure 2 and Figure 3.

The results of the computations involving this assumption are presented in Figure 6. The full circles in Figure 6a refer again to experimental data reported in [73,74], and data shown by crosses are the numerically calculated nucleation rates obtained by solving the set of kinetic equations, Equations (14)–(22). The values of the surface tension and the Tolman parameter were chosen as $\sigma_{\infty }=0.21178\;J/m^{2}$ and $\delta =0.215d_{0}$ to yield the best fit with the experimental data at temperatures, $T>T_{max}$, above the maximum of the steady-state nucleation rate. The full blue line shows the theoretical curve obtained via Equation (27), when in the analytical expression, self-consistency corrections are accounted for. The dashed line in Figure 6b is calculated, again, via the standard approach of CNT, utilizing Equation (25) with the same values of σ(T). Furthermore, in this case, the analytical expressions for the steady-state nucleation rate lead to an agreement with the results based on the set of computations, but only when self-consistency corrections are accounted for. If such corrections are omitted, then this may lead in the present case again to errors up to 7–8 orders of magnitude in the value of the steady-state nucleation rate.

#### 3.2.3. Account of a Curvature or Size Dependence of the Surface Tension for Sub- and Super-Critical Clusters

In the third approach, we assume here that the curvature dependence of the surface tension of critical clusters is given, again, by the Tolman equation, Equation (38), with the same values of the surface tension for planar interfaces and the Tolman parameter ($\sigma_{\infty }=0.21178\;J/m^{2}$, $\delta =0.215d_{0}$) as used in Figure 6. For critical clusters, the values of σ will be, consequently, the same as employed in the second set of computations resulting in Figure 6. However, we will assume now that for any value of temperature, the dependence of the surface tension on cluster size is also given by an equation of the form of Equation (38) as:

(39) $$\sigma \left(R\right)=\frac{\sigma_{\infty }}{1+\frac{2\delta }{R}}\;,\;\sigma_{\infty }=\sigma \left(R_{c}\to \infty \right)\;.$$

With this assumption, we get not only reasonable values for the surface tension of critical clusters, but also the correct limiting value for the surface tension for liquid-crystal coexistence at planar interfaces. In addition, we also obtain physically reasonable values for the surface tension for very small crystallites (σ(R→0)=0; see, e.g., [28,35]).

**Figure 7:**
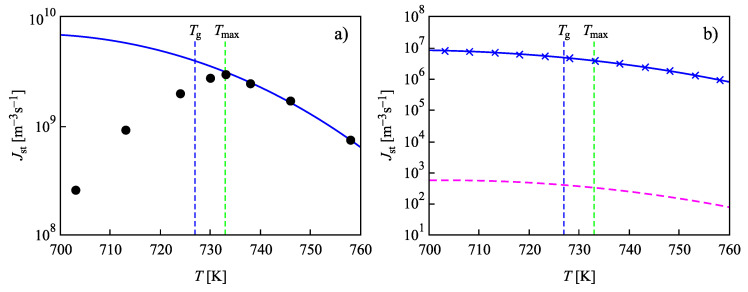
Nucleation rates for L2S versus temperature: For any temperature, the surface tension of the crystallites is assumed to obey the Tolman equation, $\sigma \left(R\right)=\sigma_{\infty }/\left(1+\left(2\delta /R\right)\right)$. The values of $\sigma_{\infty }=0.21178\;J/m^{2}$ and $\delta =0.215d_{0}$ are the same as in Figure 6, resulting in the same values of $\sigma (R_{c})$ as in Figure 6 provided Equation (2) or (9) are employed for the determination of the critical cluster size. The diffusion coefficient is described by Equation (20) with $D_{0}=1.18\times 10^{16}\;m^{2}/s$ and $E_{D}=8.864\times 10^{-19}\;J$. The filled circles in (**a**) denote experimental data given in [73,74], and the blue line in (**a**) is obtained via Equation (27) determining the parameters of the critical clusters via Equations (3) and (9). In (**b**), the blue solid line shows the theoretical curve obtained via the modification of Equation (27) and the dashed line via the modification of Equation (25). The modifications are obtained via Equation (23) using Equations (40)–(43) as explained in the text. The other parameters are specified in the captions to Figure 2 and Figure 3.

Since for critical clusters, the value of the surface tension is the same as in the computations resulting in Figure 6, one could expect similar results utilizing Equations (25) and (27) for the interpretation of the computations performed with this assumption via Equations (14)–(22). In particular, accounting for self-consistency corrections, one could expect that the analytical results for the temperature dependence of the steady-state nucleation rate are described, as in Figure 6, by the full blue curve in Figure 7a. However, comparison of the results given in Figure 6b and Figure 7b shows strong deviations. Consequently, some modifications have to be introduced here to reach an agreement of analytical expressions with the numerical computations. The reason for the necessity of the introduction of such corrections consists of the following circumstances.

As far as the surface tension has the same value independent of cluster size (first and second set of computations described above), the expressions for $n_{c}$ and the work of critical cluster formation remain the same, when Equations (7) and (8) are utilized, as in the correct approach. They are determined by Equations (3) and (9). However, accounting for a curvature dependence of the surface tension and using Equations (7) and (8) as the basic ones in the computations, we have to modify also Equations (25) and (27) in comparing analytical results and the results of the numerical computations. We have to assign different values to the critical cluster size and the work of critical cluster formation as compared to Equations (3) and (9) and have to modify also the expression for the Zeldovich factor. For the critical cluster size, we obtain instead of Equation (2):

(40) $$R_{c}=\frac{2\sigma (R_{c})}{\Delta g+{\left.\frac{\partial \sigma (R)}{\partial R}\right|}_{R=R_{c}}}\;,\;\Delta G_{c}=\frac{1}{3}\sigma A_{c}+V_{c}{\left.\frac{\partial \sigma (R)}{\partial R}\right|}_{R=R_{c}}$$

or explicitly for the critical cluster size, $R_{c}$ [103]:

(41) $$R_{c}=\frac{\left(\sigma_{\infty }-2\Delta g\delta \right)+{\left(\sigma_{\infty }^{2}+2\Delta g\delta \sigma_{\infty }\right)}^{1/2}}{\Delta g}\;.$$

With:

(42) $$\frac{4\pi }{3}{\left(\frac{R_{c}}{d_{0}}\right)}^{3}=n_{c}\;,\;\gamma =\Omega d_{0}^{2}\sigma \;,\;\Omega =4\pi {\left(\frac{3}{4\pi }\right)}^{2/3}\;,$$

we easily get then the relations for $n_{c}$, $\Delta G_{c}\left(n_{c}\right)$, and the Zeldovich factor. The Zeldovich factor, defined via Equation (24), can be written in this case as:

(43) $$\Gamma_{Z}=\sqrt{-\frac{1}{2\pi k_{B}T}{\left.\left(\frac{2\gamma }{9n_{c}^{4/3}}-\frac{4}{3n_{c}^{1/3}}\frac{\partial \gamma }{\partial n}-n_{c}^{2/3}\frac{\partial^{2}\gamma }{\partial n^{2}}\right)\right|}_{n=n_{c}}}\;.$$

Proceeding in the same way as described above, we arrive from Equation (23) at the modifications appropriate for this case of Equations (25) and (27) employed then in the computations and leading to the curves shown in Figure 7b. As is evident, accounting appropriately for such modifications, the results obtained via numerical computations coincide with the appropriate modification of Equation (27). Furthermore, in this third set of computations, self-consistency corrections are absolutely essential to reach an agreement of numerical computations and analytical equations. For the considered case, deviations between results obtained accounting for self-consistency corrections with the standard approach reach four orders of magnitude. The deviations are smaller as compared to previously considered cases here. The reason is that the value of ΔG(1) decreases. Anyway, self-consistency corrections retain their importance also in cases when a curvature dependence of the surface tension is accounted for as described above.

**Figure 8:**
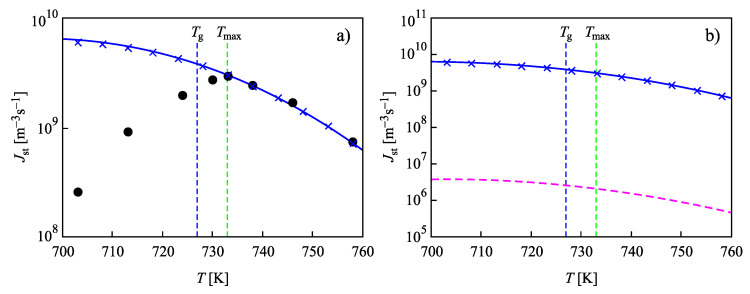
Nucleation rates for L2S versus temperature: For any temperature, the surface tension of the crystallites is assumed to obey the Tolman equation, $\sigma \left(R\right)=\sigma_{\infty }/\left(1+\left(2\delta /R\right)\right)$. The values of $\sigma_{\infty }$ and δ are chosen here as $\sigma_{\infty }=0.2233\;J/m^{2}$ and $\delta =0.341d_{0}$ to yield the best fit of numerical calculation with the experimental data at $T>T_{max}$. The diffusion coefficient is described by Equation (20) with $D_{0}=1.18\times 10^{16}\;m^{2}/s$ and $E_{D}=8.864\times 10^{-19}\;J$. The filled circles in (**a**) denote experimental data given in [73,74], and the crosses are the numerically calculated nucleation rates. The solid blue lines in (**a**,**b**) show the theoretical curve obtained via the modifications of Equation (27) accounting appropriately of self-consistency corrections and the dashed line in (**b**) results from the modification of Equation (25) (employing in Equation (23) the relations given by Equations (40)–(43)) with the same expression for σ(R). The other parameters are specified in the captions to Figure 2 and Figure 3.

To reach in such an approach an agreement with experimental data, we have to change the values of the two parameters, $\sigma_{\infty }$ and δ, in the Tolman equation. The results are presented in Figure 8. As is evident, with such slightly modified values of the parameters, an agreement both with experimental data and the results of numerical computations can be reached.

**Figure 9:**
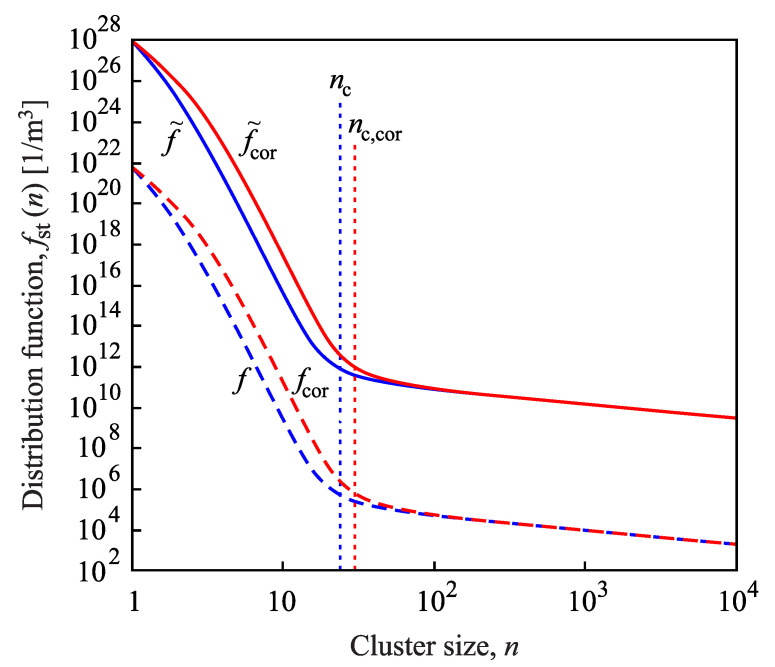
Steady-state cluster-size distribution computed via Equation (32) for T=733K. The solid lines show the theoretical curves $\tilde{f}$ and ${\tilde{f}}_{cor}$ obtained with $J_{st}$ computed via Equation (27), for $\tilde{f}$ with $\sigma =0.1703J/m^{2}$. For ${\tilde{f}}_{cor}$, σ=σ(R) as described by Equation (39), and $J_{st}$ is computed employing the corrections described by Equations (41)–(43). The dashed line *f* is calculated via the standard approach of CNT, utilizing Equation (25) with the same σ, and $f_{cor}$ for σ=σ(R). The other parameters are specified in the captions to Figure 2 and Figure 3.

Finally, in Figure 9, the dependence of the course of the steady-state cluster-size distribution on the chosen value of the steady-state nucleation rates is illustrated. Details can be found in the figure caption. As already concluded in connection with Figure 3, only if self-consistency corrections are appropriately accounted for, then Equations (32) and (33) lead to correct predictions for the steady-state cluster-size distribution.

## 4. Summary of Results and Discussion

Self-consistency corrections are commonly omitted in the theoretical analysis of crystal nucleation assuming that they have a negligible effect. We show here that this common belief is, in general, misleading.

The mentioned common belief is partly true, but only with respect to the theoretical modeling of crystal nucleation and growth based on the set of kinetic equations, Equations (14) and (15). The origin of this independence and the results of numerical solution of the set of kinetic equations on self-consistency corrections consists of the form of the relation between the coefficients of aggregation and dissolution, Equation (22) or (26). These relations are identical for both cases and lead, consequently, to the same results independent of whether a self-consistency correction is introduced or not.

However, the analytical expressions for the steady-state nucleation rate, Equation (25) or Equation (27), and the steady-state cluster size distribution, Equations (32) and (33), are significantly affected by such self-consistency corrections. These corrections are, as a rule, not negligible and, as shown, may lead to differences in the values of the steady-state nucleation rate, reaching 20 orders of magnitude. Since $\Delta \tilde{G}\left(n\right)=\Delta G\left(n\right)-\Delta G\left(1\right)<\Delta G\left(n\right)$ holds, self-consistency corrections result in higher values of the theoretical estimates of the steady-state nucleation rate as compared with the standard approach employed as a rule so far in CNT.

Applications of CNT to the interpretation of experimental nucleation data show that, as far as the capillarity approximation is employed, theoretical estimates utilizing the commonly employed relations frequently lead to too low values of the steady-state nucleation rate as compared with experimental data [2,4,5,9,33,100,101,104]. Consequently, self-consistency corrections result in an improvement of the theoretical predictions by the analytical equations with experimental data on steady-state nucleation rates. As demonstrated in the present analysis, self-consistency corrections retain their importance also in cases when a temperature or curvature dependence of the surface tension is accounted for.

Of course, one can describe experimental data by the standard analytical expressions not introducing a self-consistency correction. This is possible if one uses the surface tension and the size parameter as fitting quantities (one can include here also the diffusion coefficient not having independent data for it). Such an approach is possible in the description of crystal nucleation since the values of solid-liquid surface tension even for planar interfaces cannot be directly measured with the accuracy required for the treatment of nucleation. Here, we can even succeed to a large degree using the capillarity approximation with the consequence that surface tension and the size parameter get values that are physically senseless. In addition, one obtains values for the pre-exponential term in the expression for the steady-state nucleation rate that are in conflict with the theory underlying it. However, as soon as the condition ΔG(n)≫ΔG(1) is not fulfilled, modeling of the nucleation-growth process by the system of kinetic equations will lead to different results for the steady-state nucleation rate as compared with the analytical relations using the same parameters, resulting in the internal inconsistency of the theory.

The steady-state cluster-size distribution, described by Equation (32), is directly proportional to the steady-state nucleation rate. For this reason, Equation (32) leads to correct theoretical results for the steady-state cluster-size distribution also only if self-consistency corrections are introduced into the description.

In the present analysis, we considered homogeneous nucleation. The analytical relations for the steady-state nucleation rate describing heterogeneous nucleation can be obtained by the respective relations for homogeneous nucleation appropriately accounting for the decrease of the work of critical cluster formation caused by different factors catalyzing nucleation. The work of critical cluster formation in heterogeneous nucleation, $W_{c}^{het}$, can be expressed generally as $W_{c}^{het}=W_{c}^{hom}\Phi$, where $W_{c}^{hom}$ is the work of critical cluster formation for homogeneous nucleation at the given conditions and Φ≤1 describes the catalytic effect on nucleation of the particular nucleation cores considered [2,4,5,60]. Consequently, self-consistency corrections can be considered to be of major significance also for the correct treatment of heterogeneous nucleation.

CNT is till now the most widely applied tool for the interpretation of nucleation processes in many fields. For this reason, the analysis of the effect of self-consistency corrections in the theoretical modeling of nucleation is of outstanding importance not only for the particular case of crystallization analyzed here, but far beyond. Quite generally, as far as relations of the above given form can be considered as a reasonable approximation also for other kinds of phase formation like condensation, boiling, or segregation in solutions, the analysis performed here is of direct relevance also for these applications. This conclusion holds for sure with respect to the application of Equation (1) for the steady-state nucleation rate and Equation (32) for the steady-state cluster-size distribution. In the analysis of these alternative kinds of phase formation, its application may be even more important. In crystal nucleation, the surface tension cannot be measured directly with the precision required for an application to nucleation. For this reason, it is widely used as a fit parameter frequently without posing the question to what extent the results of such a fit are reasonable or not. In applications to boiling, condensation, or segregation, the situation is different: here, the value of the surface tension for equilibrium coexistence at planar interfaces can be experimentally measured. Consequently, the question arises whether, indeed as generally done, deviations between theoretical predictions and experimental data can be removed only via accounting for a curvature dependence of the surface tension or whether self-consistency corrections may be of even more importance. The first works in this direction have been done [2,70,71,80,105,106,107]; however, a detailed comprehensive analysis we consider as missing so far.

Self-consistency corrections do not allow us to resolve another problem in the description of crystal nucleation: the deviations of theoretical predictions and experimental data on steady-state nucleation rates for temperatures below the temperature, $T_{max}$, corresponding to the maximum of the steady-state nucleation rate or, equivalently, below the glass transition temperature, $T_{g}$, defined in line with the proposal by Tammann [89] (see Figure 4, Figure 5, Figure 6, Figure 7 and Figure 8). Both temperatures, $T_{max}$ and $T_{g}$, are directly correlated, and they are close to each other [7,108,109]. It is sometimes (incorrectly) stated that crystallization is prohibited for temperatures below $T_{g}$. Indeed, near $T_{g}$, the viscosity becomes very large or (avoiding the application of the Stokes–Einstein–Eyring relation) the diffusion coefficients governing nucleation and growth become very small. Consequently, the characteristic time-scales required for crystal nucleation and growth become very large, and crystallization may not be observed if the times of measurement are too short. Anyway, crystal nucleation may occur also below $T_{g}$. However, the description of the crystal nucleation rate below $T_{g}$, respectively, $T_{max}$, remains an unresolved puzzle. Deviations of theoretical steady-state nucleation rates from experimental data for temperatures below the temperature of maximum steady-state nucleation rates are still unexplained. In this temperature range, additional features have to be incorporated into the model of crystal nucleation accounting correctly for the interplay of crystal nucleation and glass transition [2,5,6,7,23,24,25,27,72,110]. Such additional factors could be (i) the evolution of elastic stresses and stress relaxation [4,5,27,49,50], (ii) variations of the size of the structural units in the melt responsible for crystallization [91], (iii) the effect of the spatially heterogeneous structure of glass-forming liquids on crystal nucleation [92], (iv) deviations of the bulk state parameters of the critical clusters from the respective macroscopic properties of the newly evolving crystalline phases [5,9,22,23,24,25,104] (this feature is expected to be of significant importance also at temperatures above $T_{max}$ or $T_{g}$), and (v) the interplay of relaxation and crystal nucleation [6,7,25,27,102,110,111,112]. Of course, also in such generalizations, self-consistency corrections have to be accounted for in order to yield an agreement of numerical computations based on Equations (14) and (15) and the analytical expressions, Equations (27) and (32), and both of them with experimental data. The same statement is valid in the applications of some of these concepts to nucleation in other types of phase formation like condensation, boiling, or segregation.

Summarizing the results of the present paper, we conclude: The theoretical predictions of the analytical relations for the steady-state nucleation rate and the steady-state cluster-size distribution depend significantly on the self-consistency correction. Only in the case when these corrections are taken into account, the analytical results coincide with the results of the numerical solution of the set of kinetic equations describing nucleation and growth. In particular, neglecting this self-consistency correction overestimates the work of critical cluster formation and leads, consequently, to far too low values of the theoretical predictions of the steady-state nucleation rates. For the system studied here as a typical example (lithium disilicate, ${\mathrm{Li}}_{2}O\cdot 2{\mathrm{SiO}}_{2}$), the resulting deviations from the correct values may reach 20 orders of magnitude. Consequently, neglecting self-consistency corrections may result in severe errors in the interpretation of experimental data if the analytical relations for the steady-state nucleation rate or steady-state cluster-size distribution are employed.

## References

[1] Höland W., Beall G.H. (2019). Glass-Ceramic Technology.

[2] Kelton K.F., Greer A.L. (2010). Nucleation in Condensed Matter: Applications in Materials and Biology.

[3] Gusak A.M. (2009). Diffusion-Controlled Solid State Reactions.

[4] Gutzow I.S., Schmelzer J.W.P. (1995). The Vitreous State: Thermodynamics, Structure, Rheology, and Crystallization.

[5] Gutzow I.S., Schmelzer J.W.P. (2013). The Vitreous State: Thermodynamics, Structure, Rheology, and Crystallization.

[6] Schick C., Zhuravlev E., Androsch R., Wurm A., Schmelzer J.W.P., Schmelzer J.W.P. (2014). Influence of Thermal Prehistory on Crystal Nucleation and Growth in Polymers. Glass: Selected Properties and Crystallization.

[7] Schick C., Androsch R., Schmelzer J.W.P. (2017). Topical Review: Homogeneous crystal nucleation in polymers. J. Phys. Condens. Matter.

[8] Wilde G., Schmelzer J.W.P. (2014). Early Stages of Crystal Formation in Glass-forming Metallic Alloys. Glass: Selected Properties and Crystallization.

[9] Fokin V.M., Zanotto E.D., Yuritsyn N.S., Schmelzer J.W.P. (2006). Homogeneous Crystal Nucleation in Silicate Glasses: A Forty Years Perspective. J. Non-Cryst. Solids.

[10] Skripov V.P., Koverda V.P. (1984). Spontaneous Crystallization of Undercooled Liquids.

[11] Debenedetti P. (1996). Metastable Liquids: Concepts and Principles.

[12] Skripov V.P., Faizullin M.Z. (2006). Crystal-Liquid-Gas Phase Transitions and Thermodynamic Similarity.

[13] Morey G.W. (1938). The Properties of Glass.

[14] Olovsson I. (2017). Wonders of Water: The Hydrogen Bond in Action.

[15] Markov I. (2002). Crystal Growth for Beginners: Fundamentals of Nucleation, Crystal Growth, and Epitaxy.

[16] Feigelson R.S. (2004). 50 Years Progress in Crystal Growth: A Reprint Collection.

[17] Earle S. (2019). Physical Geology.

[18] Schmelzer J.W.P., Schmelzer J.W.P., Röpke G., Priezzhev V.B. (2002). Micro- and Nanostructures: A Little Picture Book. Nucleation Theory and Applications.

[19] Zanotto E.D. (2013). Crystal in Glass: A Hidden Beauty.

[20] Gutzow I., Kashchiev D., Avramov I. (1985). Nucleation and Crystallization in Glass-forming melts: Old problems and new questions. J. Non-Cryst. Solids.

[21] Schmelzer J.W.P., Abyzov A.S. (2007). Generalized Gibbs’ Approach to the Thermodynamics of Heterogeneous Systems and the Kinetics of First-Order Phase Transitions. J. Eng. Thermophys..

[22] Schmelzer J.W.P., Fokin V.M., Abyzov A.S., Zanotto E.D., Gutzow I. (2010). How Do Crystals Form and Grow in Glass-Forming Liquids: Ostwald’s Rule of Stages and Beyond. Int. J. Appl. Glass Sci..

[23] Johari G., Schmelzer J.W.P., Schmelzer J.W.P. (2014). Crystal Nucleation and Growth in Glass-forming Systems: Some New Results and Open Problems. Glass: Selected Properties and Crystallization.

[24] Schmelzer J.W.P., Abyzov A.S., Fokin V.M. (2016). Crystallization of glass: What we know, what we need to know. Int. J. Appl. Glass Sci..

[25] Schmelzer J.W.P., Abyzov A.S. (2018). Crystallization of glass-forming melts: New answers to old questions. J. Non-Cryst. Solids.

[26] Schottelius A., Mambretti F., Kalinin A., Beyersdorff B., Rothkirch A., Goy C., Müller J., Petridis N., Ritzer M., Trinter F. (2020). Crystal growth rates in supercooled atomic liquid mixtures. Nat. Mater..

[27] Schmelzer J.W.P., Schick C., Ezquerra T.A., Nogales A. (2020). General concepts of crystallization: Some recent results and possible future developments. Dielectrics and Crystallization.

[28] Band W. (1939). Dissociation Treatment of Condensing Systems. J. Chem. Phys..

[29] Oxtoby D.W. (2003). Crystal nucleation in simple and complex fluids. Philos. Trans. R. Soc. A Math. Phys. Eng. Sci..

[30] Oxtoby D.W. (2006). Nucleation of Crystals from the Melt. Ann. N. Y. Acad. Sci..

[31] Asta M., Beckermann C., Karma A., Kurz W., Napolitano R., Plapp M., Purdy G., Rappaz M., Trivedi R. (2009). Solidification microstructures and solid-state parallels: Recent developments, future directions. Acta Mater..

[32] Granasy L., Toth G.I., Warren J.A., Podmaniczky F., Tegze G., Ratkai L., Pusztai T. (2019). Phase-field modeling of crystal nucleation in undercooled liquids—A review. Prog. Mater. Sci..

[33] Baidakov V.G., Schmelzer J.W.P. (2014). Crystallization of Undercooled Liquids: Results of Molecular Dynamics Simulations. Glass: Selected Properties and Crystallization.

[34] Schmelzer J.W.P., Abyzov A.S., Ferreira E.B., Fokin V.M. (2019). Curvature dependence of the surface tension and crystal nucleation in liquids. Int. J. Appl. Glass Sci..

[35] Schmelzer J.W.P., Abyzov A.S., Baidakov V.G. (2019). Entropy and the Tolman Parameter in Nucleation Theory. Entropy.

[36] Gibbs J.W. (1875–1878). On the Equilibrium of Heterogeneous Substances. Trans. Conn. Acad. Sci..

[37] Gibbs J.W. (1928). On the Equilibrium of Heterogeneous Substances. Thermodynamics.

[38] Van der Waals J.D., Kohnstamm P. (1908). Lehrbuch der Thermodynamik (English: Textbook on Thermodynamics).

[39] Rowlinson J.S. (1979). Translation of J. D. van der Waals’ “The Thermodynamic Theory of Capillarity Under the Hypothesis of a Continuous Variation of Density”. J. Stat. Phys..

[40] Einstein A. (1906). Zur Theorie der Brownschen Bewegung (Engl: On the theory of Brownian motion). Ann. Der Phys..

[41] Einstein A. (1910). Theorie der Opaleszenz von homogenen Flüssigkeiten und Flüssigkeitsgemischen in der Nähe des kritischen Zustandes (Engl: The Theory of the Opalescence of Homogeneous Fluids and Liquid Mixtures near the Critical State). Ann. Der Phys..

[42] Landau L.D., Lifschitz E.M. (1969). Statistische Physik (Engl.: Statistical Physics).

[43] Volmer M., Weber A. (1926). Keimbildung in übersättigten Gebilden (Engl.: Nucleation in supersaturated samples). Z. Phys. Chem..

[44] Schmelzer J.W.P., Abyzov A.S. (2016). Crystallization of glass-forming liquids: Thermodynamic driving force. J. Non-Cryst. Solids.

[45] Schmelzer J.W.P., Pascova R., Möller J., Gutzow I. (1993). Surface Induced Devitrification of Glasses: The Influence of Elastic Strains. J. Non-Cryst. Solids.

[46] Schmelzer J.W.P., Möller J., Gutzow I., Pascova R., Müller R., Pannhorst W. (1995). Surface-energy and Structure Effects on Surface Crystallization. J. Non-Cryst. Solids.

[47] Schmelzer J.W.P., Müller R., Möller J., Gutzow I.S. (2002). Elastic Stresses, Stress Relaxation, and Crystallization: Theory. Phys. Chem. Glas..

[48] Schmelzer J.W.P., Müller R., Möller J., Gutzow I.S. (2003). Theory of Nucleation in Viscoelastic Media: Application to Phase Formation in Glassforming Melts. J. Non-Cryst. Solids.

[49] Fokin V.M., Zanotto E.D., Schmelzer J.W.P., Potapov O.V. (2005). New Insights on the Thermodynamic Barrier for Nucleation in Lithium Disilicate Glass. J. Non-Cryst. Solids.

[50] Abyzov A.S., Fokin V.M., Rodrigues A.M., Zanotto E.D., Schmelzer J.W.P. (2016). The effect of elastic stresses on the thermodynamic barrier for crystal nucleation. J. Non-Cryst. Solids.

[51] Slezov V.V., Schmelzer J.W.P. (1994). Kinetics of formation of a phase with a definite stoichiometric composition. J. Phys. Chem. Solids.

[52] Slezov V.V., Schmelzer J.W.P. (1998). Comments on Nucleation Theory. J. Phys. Chem. Solids.

[53] Slezov V.V., Schmelzer J.W.P. (2002). Kinetics of formation of a phase with an arbitrary stoichiometric composition in a multi-component solid solution. Phys. Rev. E.

[54] Schmelzer J.W.P. (2008). Crystal nucleation and growth in glass-forming melts: Experiment and theory. J. Non-Cryst. Solids.

[55] Schmelzer J.W.P. (2010). On the determination of the kinetic pre-factor in classical nucleation theory. J. Non-Cryst. Solids.

[56] Blanc W., Martin I., Francois-Saint-Cyr H., Bidault X., Chaussedent S., Hombourger C., Lacomme S., le Coustumer P., Neuville D.R., Larson D.J. (2019). Compositional Changes at the Early Stages of Nanoparticles Growth in Glasses. J. Phys. Chem. C.

[57] Abyzov A.S., Schmelzer J.W.P., Kovalchuk A.A., Slezov V.V. (2010). Evolution of Cluster Size-Distributions in Nucleation-Growth and Spinodal Decomposition Processes in a Regular Solution. J. Non-Cryst. Solids.

[58] Abyzov A.S., Schmelzer J.W.P. (2014). Kinetics of segregation processes in solutions: Saddle point versus ridge crossing of the thermodynamic potential barrier. J. Non-Cryst. Solids.

[59] Farkas L. (1927). Keimbildungsgeschwindigkeit in übersättigten Dämpfen (Engl.: Nucleation rate in supersaturated vapors). Z. Phys. Chem..

[60] Volmer M. (1939). Kinetik der Phasenbildung (English: Kinetics of Phase Formation).

[61] Kaischew R., Stranski I.N. (1934). Zur Theorie der linearen Kristallisationsgeschwindigkeit (Engl: On the theory of the linear rate of crystallization). Z. Phys. Chem. A.

[62] Becker R., Döring W. (1935). Kinetische Behandlung der Keimbildung in übersättigten Dämpfen (Engl.: Kinetic treatment of nucleation in supersaturated vapors). Ann. Der Phys..

[63] Frenkel Y.I. (1946). The Kinetic Theory of Liquids.

[64] Zeldovich Y.B. (1942). On the Theory of New Phase Formation: Cavitation. Sov. Phys. JETP.

[65] Turnbull D., Fisher J.C. (1949). Rate of Nucleation in Condensed Systems. J. Chem. Phys..

[66] Ulbricht H., Schmelzer J.W.P., Mahnke R., Schweitzer F. (1988). Thermodynamics of Finite Systems and the Kinetics of First-Order Phase Transitions.

[67] Schmelzer J.W.P., Schmelzer J.W.P., Röpke G., Priezzhev V.B. (1999). Comments on Curvature Dependent Surface Tension and Nucleation Theory. Nucleation Theory and Applications.

[68] Schmelzer J.W.P., Boltachev G.S., Baidakov V.G. (2006). Classical and Generalized Gibbs’ Approaches and the Work of Critical Cluster Formation in Nucleation Theory. J. Chem. Phys..

[69] Schmelzer J.W.P. (2019). Application of the Nucleation Theorem to Crystallization of Liquids: Some General Theoretical Results. Entropy.

[70] Wu D.T., Ehrenreich H., Spaepen F. (1997). Nucleation Theory. Solid State Physics.

[71] Ford I.J. (2004). Statistical mechanics of nucleation: A review. Proc. Inst. Mech. Eng. Part C J. Mech. Eng. Sci..

[72] Bartels J., Lembke U., Pascova R., Schmelzer J., Gutzow I. (1991). Evolution of Cluster Size Distributions in Nucleation and Growth Processes. J. Non-Cryst. Solids.

[73] Fokin V.M. (1980). Investigation of Stationary and Non-Stationary Crystal Nucleation Rates in Model Glasses of Stoichiometric Composition Li_2_O·2SiO_2_ and 2Na_2_O·CaO·3SiO_2_. Ph.D. Thesis.

[74] Fokin V.M., Kalinina A.M., Filipovich V.N. (1981). Nucleation in silicate glasses and effect of preliminary heat treatment on it. J. Cryst. Growth.

[75] Slezov V.V., Schmelzer J.W.P., Schmelzer J.W.P., Röpke G., Priezzhev V.B. (1999). Kinetics of Nucleation-Growth Processes: The First Stages. Nucleation Theory and Applications.

[76] Schmelzer J.W.P., Slezov V.V., Röpke G., Schmelzer J., Schmelzer J.W.P., Röpke G., Priezzhev V.B. (1999). Shapes of Cluster Size Distributions Evolving in Nucleation—Growth Processes. Nucleation Theory and Applications.

[77] Slezov V.V. (2009). Kinetics of First-Order Phase Transitions.

[78] Von Smoluchowski M. (1917). Versuch einer mathematischen Theorie der Koagulationskinetik kolloider Lösungen (Engl.: Attempt of a mathematical theory of the coagulation kinetics of colloidal solutions). Z. Phys. Chem..

[79] Binder K., Stauffer D. (1976). Statistical theory of nucleation, condensation, and coagulation. Adv. Phys..

[80] Wyslouzil B.E., Wilemski G. (1995). Binary nucleation kinetics. II. Numerical solution of the birth-death equations. J. Chem. Phys..

[81] Clouet E., Furrer D.U., Semiatin S.L. (2009). Modeling of Nucleation Processes. Fundamentals of Modeling for Metals Processing.

[82] Schmelzer J.W.P., Ulbricht H. (1987). Thermodynamics of Finite Systems and the Kinetics of First-Order Phase Transitions. J. Colloid Interface Sci..

[83] Abyzov A.S., Schmelzer J.W.P. (2007). Nucleation versus Spinodal Decomposition in Confined Binary Solutions. J. Chem. Phys..

[84] Schmelzer J.W.P., Abyzov A.S. (2011). Thermodynamic analysis of nucleation in confined space: Generalized Gibbs’ approach. J. Chem. Phys..

[85] Slezov V.V., Schmelzer J.W.P., Abyzov A.S., Schmelzer J.W.P. (2005). A New Method of Determination of the Coefficients of Emission in Nucleation Theory. Nucleation Theory and Applications.

[86] Gutzow I., Schmelzer J.W.P., Dobreva A. (1997). Kinetics of Transient Nucleation in Glass-Forming Liquids: A Retrospective and Recent Results. J. Non-Cryst. Solids.

[87] Schmelzer J.W.P., Abyzov A.S., Baidakov V.G. (2017). Time of formation of the first supercritical nucleus, time-lag, and the steady-state nucleation rate. Int. J. Appl. Glass Sci..

[88] Schmelzer J.W.P., Abyzov A.S. (2011). On the theoretical description of nucleation in confined space. Am. Insitute Phys. Adv..

[89] Tammann G. (1933). Der Glaszustand (Engl.: The Vitreous State).

[90] Kalinina A.M., Filipovich V.N., Fokin V.M. (1980). Stationary and non-stationary crystal nucleation in a glass of 2Na_2_0·CaO·3SiO_2_ stoichiometric composition. J. Non-Cryst. Solids.

[91] Fokin V.M., Abyzov A.S., Zanotto E.D., Cassar D.R., Rodrigues A.M., Schmelzer J.W.P. (2016). Crystal nucleation in glass-forming liquids: Variation of the size of the “structural units” with temperature. J. Non-Cryst. Solids.

[92] Abyzov A.S., Fokin V.M., Yuritsyn N.S., Rodrigues A.M., Schmelzer J.W.P. (2017). The effect of heterogeneous structure of glass-forming liquids on crystal nucleation. J. Non-Cryst. Solids.

[93] Ono S., Kondo S., Flügge S. (1960). Molecular theory of surface tension in liquids. Handbuch der Physik (Encyclopedia of Physics).

[94] Renninger R.G., Hiller F.C., Bone R.C. (1981). Comment on “Self-nucleation in the sulfuric acid-water system”. J. Chem. Phys..

[95] Wilemski G. (1984). Composition of the critical nucleus in multi-component vapor nucleation. J. Chem. Phys..

[96] Schmelzer J.W.P., Mahnke R. (1986). General Formulae for the Curvature Dependence of Droplets and Bubbles. J. Chem. Soc. Faraday Trans. I.

[97] Schmelzer J.W.P. (1986). The Curvature Dependence of Surface Tension of Small Droplets. J. Chem. Soc. Faraday Trans. I.

[98] Schmelzer J.W.P., Gutzow I., Schmelzer J. (1996). Curvature Dependent Surface Tension and Nucleation Theory. J. Colloid Interface Sci..

[99] Schmelzer J.W.P., Baidakov V.G. (2016). Comment on “Simple improvements to classical nucleation models”. Phys. Rev. E.

[100] Baidakov V.G. (2007). Explosive Boiling of Superheated Cryogenic Liquids.

[101] Tolman R. (1949). The Effect of Droplet Size on Surface Tension. J. Chem. Phys..

[102] Schmelzer J.W.P., Abyzov A.S., Fokin V.M., Schick C. (2018). Kauzmann paradox and the crystallization of glass-forming melts. J. Non-Cryst. Solids.

[103] Fokin V.M., Zanotto E.D. (2000). Crystal nucleation in silicate glasses: The temperature and size dependence of crystal/liquid surface energy. J. Non-Cryst. Solids.

[104] Schmelzer J.W.P., Abyzov A.S. (2014). Comments on the thermodynamic analysis of nucleation in confined space. J. Non-Cryst. Solids.

[105] Blander M., Katz J.L. (1972). The Thermodynamics of Cluster Formation in Nucleation Theory. J. Stat. Phys..

[106] Girshick S.L., Chiu C.-P. (1990). Kinetic nucleation theory: A new expression for the rate of homogeneous nucleation from an ideal supersaturated vapor. J. Chem. Phys..

[107] Girshick S.L. (1991). Comment on: “Self-consistency correction to homogeneous nucleation theory”. J. Chem. Phys..

[108] Fokin V.M., Zanotto E.D., Schmelzer J.W.P. (2003). Homogeneous Nucleation versus Glass Transition Temperature. J. Non-Cryst. Solids.

[109] Schmelzer J.W.P., Abyzov A.S., Fokin V.M., Schick C., Zanotto E.D. (2015). Crystallization in glass-forming liquids: Effects of fragility and glass transition temperature. J. Non-Cryst. Solids.

[110] Schmelzer J.W.P., Schick C. (2012). Dependence of Crystallization Processes of Glass-forming Melts on Prehistory: A Theoretical Approach to a Quantitative Treatment. Phys. Chem. Glas. Eur. J. Glass Sci. Technol. B.

[111] Zanotto E.D., Cassar D.R. (2018). The race within supercooled liquids: Relaxation versus crystallization. J. Chem. Phys..

[112] Schmelzer J.W.P., Tropin T.V. (2018). Glass transition, crystallization of glass-forming melts, and entropy. Entropy.

